# Evaluation of the Influence of Primary and Secondary Crystal Orientations and Selected Structural Characteristics on Creep Resistance in Single-Crystal Nickel-Based Turbine Blades

**DOI:** 10.3390/ma18050919

**Published:** 2025-02-20

**Authors:** Kamil Gancarczyk, Robert Albrecht, Paweł Sułkowicz, Mirosław Szala, Mariusz Walczak

**Affiliations:** 1 Department of Materials Science, Faculty of Mechanical Engineering and Aeronautics, Rzeszow University of Technology, 12 Powstancow Warszawy Ave., 35-959 Rzeszow, Poland; 2 Institute of Materials Science, University of Silesia in Katowice, 1a 75 Pulku Piechoty St., 41-500 Chorzow, Poland; robert.albrecht@us.edu.pl; 3 Department of Manufacturing Techniques and Automation, Faculty of Mechanical Engineering and Aeronautics, Rzeszow University of Technology, 12 Powstancow Warszawy Ave., 35-959 Rzeszow, Poland; sulkowicz@prz.edu.pl; 4 Department of Materials Engineering, Faculty of Mechanical Engineering, Lublin University of Technology, 36 Nadbystrzycka St., 20-618 Lublin, Poland; m.szala@pollub.pl (M.S.); m.walczak@pollub.pl (M.W.)

**Keywords:** superalloy, crystal orientation, turbine blades, single crystal, creep resistance

## Abstract

This study evaluates the perfection of the crystal structure of single-crystal turbine blade castings made from the CMSX-4 nickel superalloy. The analysis included primary and secondary crystal orientation measurements using the Ω-scan method and the novel OD-EFG X-ray diffractometer. The selected microstructural parameters of the single crystals were also analyzed, including the assessment of stereological parameters and the degree of porosity. A creep test was performed according to standard procedures and under conditions simulating real operational environments. The model single-crystal turbine blades were manufactured using the Bridgman–Stockbarger method, with variable withdrawal rates of 1 and 3 mm/min. Heat treatment of the single-crystal castings involved solution treatment followed by double aging. The evaluation of structural perfection was carried out in three states: as-cast, after solution heat treatment, and after double aging. The crystallographic orientation of the blades was determined on both the airfoil and the root part. The study determined how crystallographic orientation and microstructural parameters influence the creep resistance of the castings. It was found that in the as-cast condition, the greatest influence on high creep strength has a small deviation of the primary and constant value of secondary crystal orientation along the height of the blade casting. After heat treatment, the highest creep resistance was obtained for the blade manufactured at a withdrawal rate at 1 mm/min.

## 1. Introduction

The development of many fields of technology is closely related to the use of single crystals, particularly in aviation, where nickel-based superalloy turbine blade castings are widely used. These materials exhibit excellent resistance to high temperatures, significant mechanical and thermal loads, and the harsh environment of hot oxidizing gases. Nickel-based single-crystal (NBSX) turbine blades are primarily used for the first- and second-stage components of aircraft engine turbines [1,2,3].

NBSX turbine blade castings consist of two primary phases in their microstructure: the γ matrix and the γ’ strengthening phase. The matrix of NBSX is formed by γ-phase crystallites, a disordered solid solution of nickel with a face-centered cubic (FCC) structure. The relative volume of γ-phase crystallites in NBSX typically ranges from V_vγ_ = 20% to 40% [4]. The γ’ phase, which serves as the strengthening phase, is an intermetallic compound Ni_3_(Al, Ti, Ta) that takes the form of cubic precipitates. It has a partially ordered structure. The relative volume of γ’ phase crystallites in NBSX usually ranges from V_vγ’_ = 60% to 80% [5]. Additionally, an eutectic mixture of γ and γ’ phases may form during the solidification process, with a relative volume in NBSX of up to 15%. This eutectic increases the brittleness and susceptibility of NBSX to cracking [6,7].

The most commonly used method for producing NBSX is the Bridgman–Stockbarger method (BSM) [8]. The solidification process of NBSX via BSM typically takes place in a vertical vacuum furnace using a pre-prepared ceramic mold. The objective of this process is to produce NBSX castings with a crystallographic orientation close to the [001] direction, aligned with the axis “z” of solidification. It has been established that the [001] orientation of the crystal significantly improves the creep resistance of the casting [1,4]. A key parameter in the BSM process is the mold withdrawal rate (w_r_), which is the primary factor governing directional solidification and the resulting microstructure of the single-crystal castings [1,3]. Increasing the w_r_ decreases the primary and secondary dendrite arm spacing (PDAS, SDAS), which in turn enhances the mechanical properties of the casting [9,10]. In industrial practice, the w_r_ is typically in the range of 1–5 mm/min [11].

The most important parameter for characterizing the perfection of the crystal structure of NBSX castings—and thus the usability of the produced single-crystal blades—is the deviation angle α_z_, which represents the angle between the crystallographic [001] direction and the axis “z” of solidification (primary crystal orientation) [12,13,14]. A large deviation reduces the creep resistance of NBSX. Another frequently used parameter is the rotation angle γ_z_, which represents deviations of the projection line of the direction vector perpendicular to the direction [001], e.g., [010] from the second reference axis “y” (secondary crystal orientation) [13]. It has been shown that the γ_z_ value often changes along the height of the casting, typically due to macroscopic defects such as mosaic blocks and small-angle boundaries in different zones of the single crystal. These defects reduce the plasticity of the material and can initiate microcracks. It has been demonstrated that the absence of defects like mosaic blocks and small-angle boundaries ensures a consistent β_z_ rotation angle along the height of the blade, from the root to the tip [5,10,15]. In the aerospace industry and the production of single-crystal turbine blades, determining the primary and secondary orientations is required because even small deviations from the ideal crystallographic orientation can significantly affect the mechanical properties of the material, especially under conditions of high temperature and operational loads. Even minor misorientations can lead to unfavorable stresses and defects, increasing the risk of fatigue cracking [3,11].

Furthermore, the shape and size of the γ’-phase precipitates also influence the creep resistance of NBSX [16]. It has been found that the best creep resistance is observed in single crystals where the γ’ phase precipitates are cuboidal shaped, with side lengths of 0.2–0.6 μm [17].

Another factor that affects the creep rate at high temperatures is the presence of shrinkage pores formed during directional solidification. These pores, which are primarily located in interdendritic regions, vary in size and relative volume, depending on the crystallization conditions. The size of the pores tends to increase with larger PDAS and SDAS values. The increase in porosity should lead to a decrease in the creep resistance of the blades [18].

NBSX castings typically undergo heat treatment, including solution treatment and aging, under reduced pressure to homogenize the dendritic microstructure and reduce chemical segregation. This treatment enhances the high-temperature strength of the castings, particularly under creep conditions. Additionally, heat treatment stabilizes the phase components of the microstructure and reduces the density of crystal defects. Solution treatment and aging also decrease or completely eliminate harmful eutectic structures in NBSX [19,20,21].

The first objective of this study was to evaluate the primary and secondary crystal orientations in model NBSX turbine blades similar to those used in aircraft engines. The crystal orientation was determined from the entire casting surface of the blades. The second aim was to analyze the influence of the primary and secondary crystal orientations of the as-cast NBSX state on creep strength. Additionally, the effects of heat treatment, consisting of solution treatment and double aging, on the microstructure, porosity, and creep strength were investigated. To achieve two research aims, the research methodology used was the same as in the quality control of blades for the aviation industry: light and electron microscopy and X-ray diffraction. A creep test was used to evaluate the blade in conditions similar to operating conditions.

## 2. Materials and Methods

The CMSX-4 superalloy, with the following chemical composition, was used for the experiments (wt.%): Cr-6.5, Co-9.0, Mo-0.6, Al-5.6, Ti-1.0, Ta-6.5, Hf-0.1, Re-3.0, W-6.0, and Ni as the matrix.

### 2.1. Manufacturing Process

**Figure 1:**
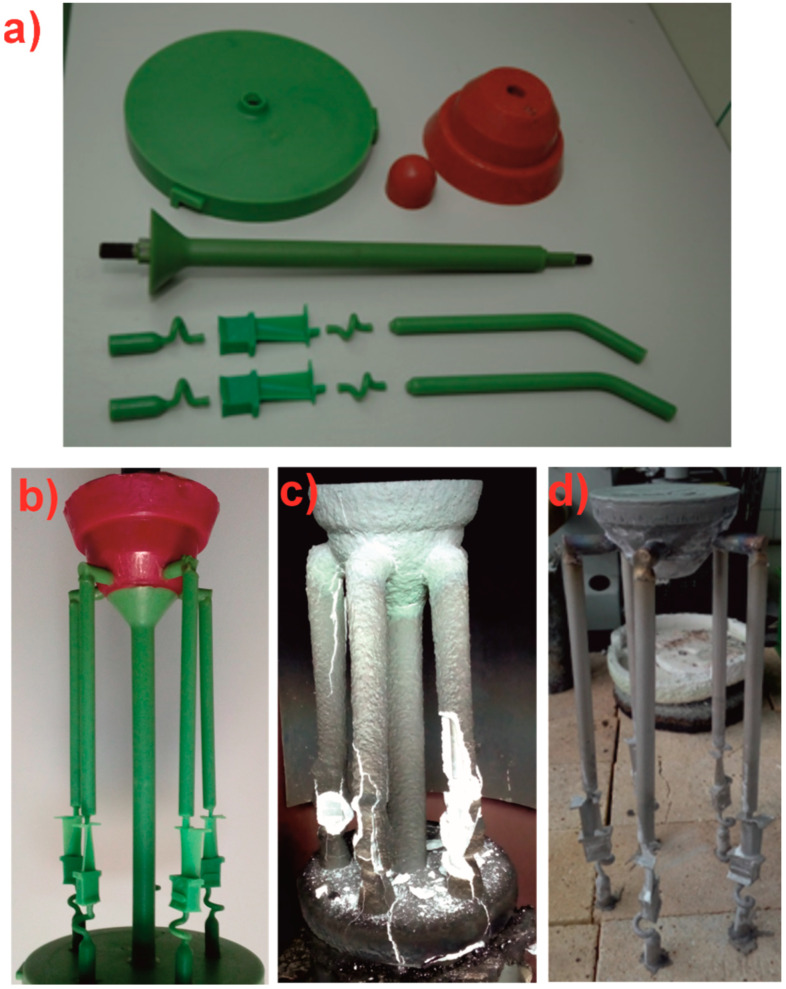
Stages of manufacturing NBSX turbine blades: (**a**) wax components, (**b**) combined wax set, (**c**) ceramic mold after the SX process, and (**d**) NBSX castings.

Wax models of rods with a diameter of 12 mm and a length of 250 mm, as well as first-stage turbine blades, were used to produce the molds (Figure 1a). The wax elements were produced using a high-pressure injection molding machine and injection molds. The following components were created: a structural mandrel, a supply system, a pouring basin, a plate, a selector, a starter, and research casting models. These elements were assembled into wax sets (Figure 1b). The wax sets were degreased in the tirisol solvent for approximately 30 s and washed twice with water. After drying, the sets were used to produce multi-layer ceramic molds. The mold consisted of nine ceramic layers applied to the wax model. The first layer, directly applied to the wax, was made of a corundum ceramic slurry and backfill. Layers 2, 3, and 4 were made of mullite ceramic slurry and backfill, while layers 5–8 used a coarser mullite backfill. The outer layer consisted of corundum ceramic slurry (Figure 1c). The mold was dried for three days to achieve the required mechanical properties.

The FCR CALDAIE autoclave was used to melt the wax model from the ceramic mold. Residual wax was burned out in an IZO chamber furnace at 760 °C. As a result, the molds with the required heat resistance and mechanical strength, were prepared for the directional solidification process of single-crystal castings. The SX casting process was carried out using a VIM 2 E–DS/SC ALD Vacuum Technologies furnace (Hanau, Germany). The ceramic mold was placed on a cooling plate and moved from the furnace’s cooling zone to the heating zone. A 2.65 kg ingot of CMSX-4 superalloy was melted in a corundum crucible under reduced pressure (~0.3 Pa). The mold was then filled with molten metal at 1520 °C. After filling, the molds were held at this temperature for 40 s and then withdrawn at a constant rate from the furnace’s heating zone to its cooling zone. Two SX casting processes were conducted at withdrawal rates of 1 mm/min and 3 mm/min. The value of the withdrawal rates was selected based on the source and is typical in terms of the range for solidification of NBSX [10,18]. The temperature gradient depending on the withdrawal rate ranged from 1.5 to 3.5 K/mm. After the process, the ceramic molds were removed, the gating system was cut off, and the castings were cleaned by sandblasting (Figure 1d).

### 2.2. Heat Treatment

**Figure 2:**
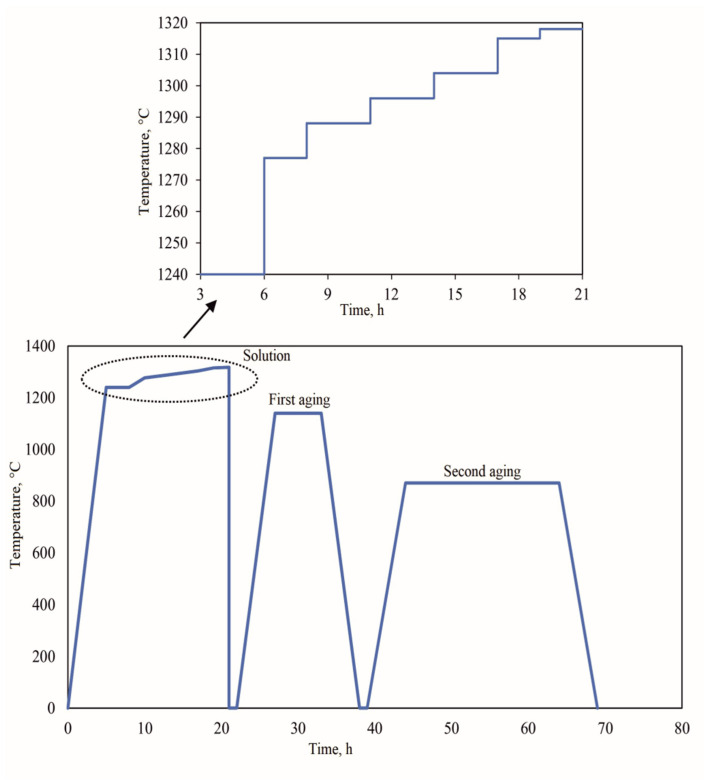
Heat treatment scheme of single-crystal nickel superalloy castings CMSX-4.

Heat treatment of the NBSX castings was performed using an ALD Mono Therm HK.446.N.20.gr vacuum furnace. The gradual heating process for solution annealing and subsequent aging was conducted under reduced pressure of 3∙× 10⁻⁴ Pa. Temperature measurements were taken using S-type platinum-sheathed thermocouples. The castings were first heated to 1240 °C and annealed for 3 h. They were then gradually heated according to the following schedule: 1277 °C for 2 h, 1288 °C for 3 h, 1296 °C for 3 h, 1304 °C for 2 h, and finally 1315 °C for 2 h, with the final solution annealing temperature of 1318 °C held for 2 h (Figure 2). The solution heat treatment was performed in an argon atmosphere. After solution treatment, the castings underwent two stages of aging. The first aging was carried out at 1140 °C for 6 h, with furnace cooling. The second aging stage was performed at 870 °C for 20 h, also with furnace cooling to room temperature (S+A) [6]. The heat treatment schedule was based on the γ’ solvus and solidus temperatures of CMSX-4, ensuring the homogenization of the microstructure without risking partial melting. The two-stage aging process (1140 °C followed by 870 °C) was optimized to achieve a uniform cubic γ’ phase morphology, critical for enhancing creep resistance.

### 2.3. Characterization

**Figure 3:**
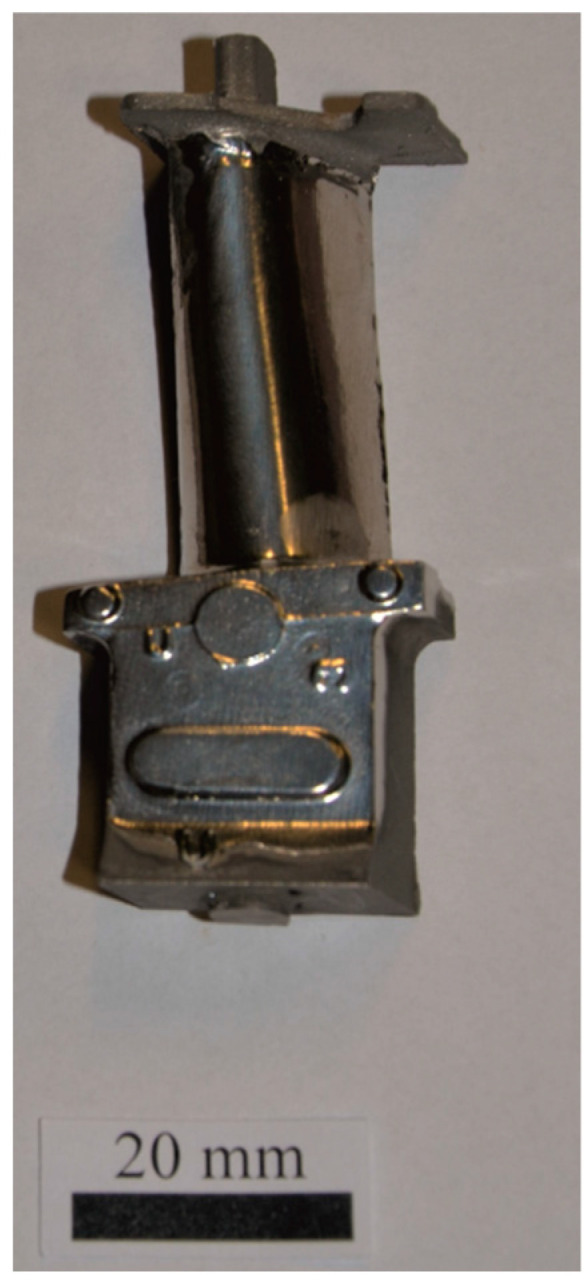
Turbine blade after electrolytic polishing.

The airfoil and root regions of the blades were electrolytically polished to determine their crystallographic orientation using an electrolyte composed of HClO_4_ (200 cm^3^) and CH_3_OH (800 cm^3^). The electrolyte temperature was maintained at −26 °C, with a current of 40 mA and a voltage of 11 V. The polishing time for the blade surfaces was 20 to 30 min (Figure 3).

Secondary dendrite arm spacing (SDAS) was measured using a Nikon EPIPHOT 300 metallographic microscope (Tokyo, Japan) equipped with a digital camera and NIS-Elements-AR image analysis software (https://www.microscope.healthcare.nikon.com/products/software/nis-elements/nis-elements-advanced-research). The SDAS values were calculated using the following formula [22]:

(1) $$\lambda_{2}=\frac{L}{\left(n-1\right)},\;[um]$$

where L is the length of the measured section and n is the number of secondary dendrite arms along that section.

The microstructure, including the γ and γ’ phases, was analyzed using a HITACHI S-3400 NII scanning electron microscope (Tokyo, Japan). Samples for SEM observation were prepared by the electro-spark cutting of the blade root. Then, the flat surface was ground with SiC sandpaper (P220-P1200) and polished using MD-Mol cloth and DP suspension diamond paste. The chemical etching of samples was conducted in a solution containing 3 g MoO^3^, 100 cm^3^ HCl, 100 cm^3^ HNO^3^ and 100 cm^3^ H_2_O. The stereological parameters of the microstructure were determined using Leica Application Suite v3.7 image analysis software. The surface area and relative volume of the γ’ precipitates were calculated, with the sum of γ and γ’ phases assumed to be 100%.

**Figure 4:**
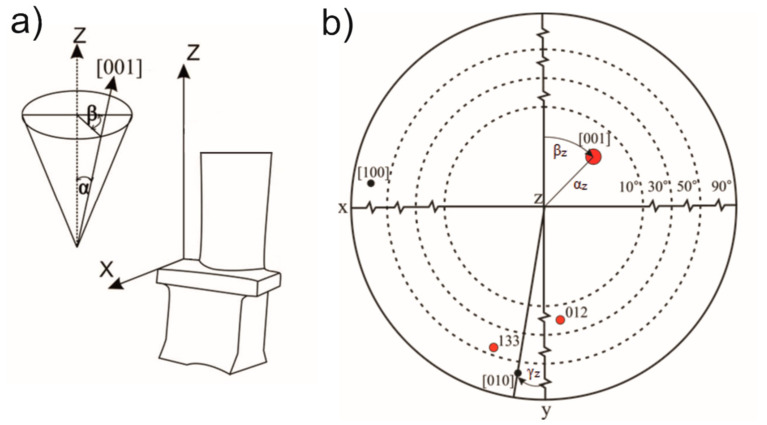
Scheme of determining the α_z_ and β_z_ angle, characterizing the perfection of the NBSX castings structure (**a**); stereographic projection of the full orientation of a single crystal (**b**).

Crystal perfection was evaluated by measuring the changes in three angles: the primary orientation angle (α_z_), which represents the angle between the [001] crystallographic direction and the blade axis “z”; the rotation angle (β_z_), which describes the rotation of the [001] direction around the casting direction; and the third orientation angle (γ_z_), which measures the deviation of the [010] direction from the reference axis (Figure 4). The crystal orientation measurements were taken using the Ω-scan method and an OD-EFG1 X-ray diffractometer [23]. A copper lamp (Cu_Kα_ = 0.154 nm), a round collimator with a diameter of 0.8 mm, was used. The voltage applied to the lamp was 40 kV, and the current was 30 mA. The Ω-scan method was selected due to its ability to measure crystallographic orientation across the entire blade surface, providing comprehensive data compared to point-based methods like EBSD or Laue diffraction. Although polishing is required to remove the casting skin, the process does not alter the crystallographic orientation, as verified in previous studies [23].

Porosity was determined from unetched metallographic microsections. Micrographs of pores were captured using a Leica DMI3000M light microscope (Wetzlar, Germany) at 50× magnification. Ten images from different areas of the sample surface (1.4 mm^2^) were analyzed. The relative volume of pores was calculated using Leica Application Suite v3.7 software.

### 2.4. Creep Testing

**Figure 5:**
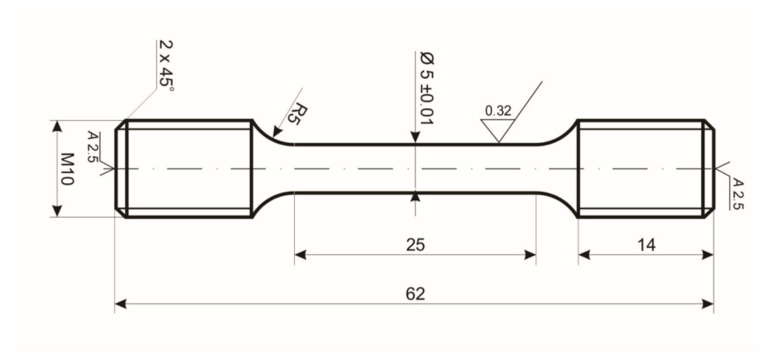
Shape and dimensions [mm] of the creep resistance test sample.

Creep tests were performed to evaluate the creep resistance of CMSX-4 single crystals. The tests were conducted on a Walter + Bai AG LFMZ-30 creep tester in accordance with ASTM E-139-11 [24], using round specimens prepared according to ASTM E8 (Figure 5) [25]. The samples were turned using a CNCNEF 600 lathe (Fermat Machinery, Radějovice, Czech Republic). Temperatures of 982 °C were adopted for the test, the S-type thermocouple—PtRh10-Pt, and the initial tensile stress σ = 151.8 MPa.

## 3. Results

### 3.1. Microstructure

**Figure 6:**
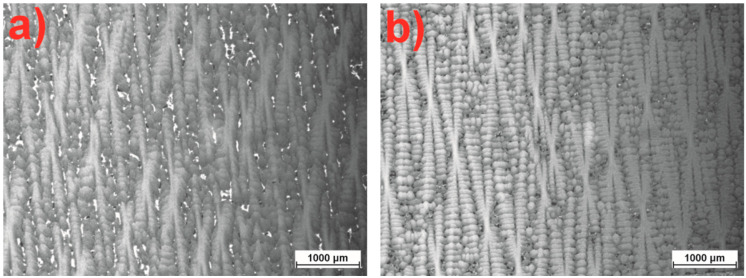
Microstructure of the CMSX-4 superalloy. Morphology of dendrites in the longitudinal section for the withdrawal rate: (**a**) 1, (**b**) 3 mm/min.

The microscopic analysis of the CMSX-4 superalloy using optical microscopy revealed that after the single-crystal (SX) process, the material exhibits a dendritic structure. It was observed that the shape of the dendrites and the distance between them change with an increase in the withdrawal rate (w_r_) during the crystallization process. Specifically, the secondary dendrite arm spacing (SDAS) decreases as the w_r_ increases within the range of 1 to 3 mm/min (Table 1). This reduction, approximately 25 μm, is significant. Thus, it can be concluded that increasing the w_r_ promotes a more refined dendritic structure (Figure 6a,b).

**Figure 7:**
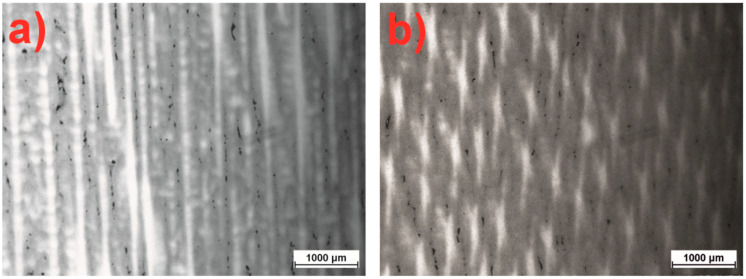
Microstructure of the CMSX-4 superalloy after heat treatment (w_r_ = 1 mm/min): (**a**) solution, (**b**) solution + double aging.

Further microscopic examinations of the dendritic morphology of the CMSX-4 nickel superalloy after heat treatment revealed the dissolution of the dendritic microstructure (Figure 7a,b). During solution heat treatment, the dendrite arms undergo substantial restructuring, aligning almost parallel to the crystallographic [001] direction. The aging process significantly alters their shape, causing the dendrites to dissolve or disappear entirely. This transformation is likely associated with the restructuring of the crystal structure, the homogenization of the chemical composition, and a shift from a single-crystal dendritic-type microstructure to a more cellular-type single-crystal structure. Thus, it can be concluded that, under the given conditions of the SX process for the CMSX-4 nickel superalloy, the castings initially exhibit a dendritic single-crystal structure. However, after heat treatment (solution heat treatment and double aging), the castings transform into a cellular single-crystal structure.

The relative volume of the γ’ phase in the CMSX-4 superalloy microstructure was determined using quantitative metallography. In the as-cast state, the volume fraction of the γ’ phase (V_vγ’_) was 64.8% for w_r_ = 1 mm/min and 64.0% for w_r_ = 3 mm/min (Table 2). It was also observed that the γ’ phase content in the interdendritic regions was 11–13% higher than in the dendritic areas, depending on the w_r_ of the single crystal. Heat treatment, specifically solution treatment, led to an increase in the γ’ phase content by 1–2.5% (Table 2). Moreover, full heat treatment (solution treatment and double aging) of CMSX-4 single crystals resulted in a similar γ’ phase content of approximately 67–68%, regardless of the w_r_.

**Figure 8:**
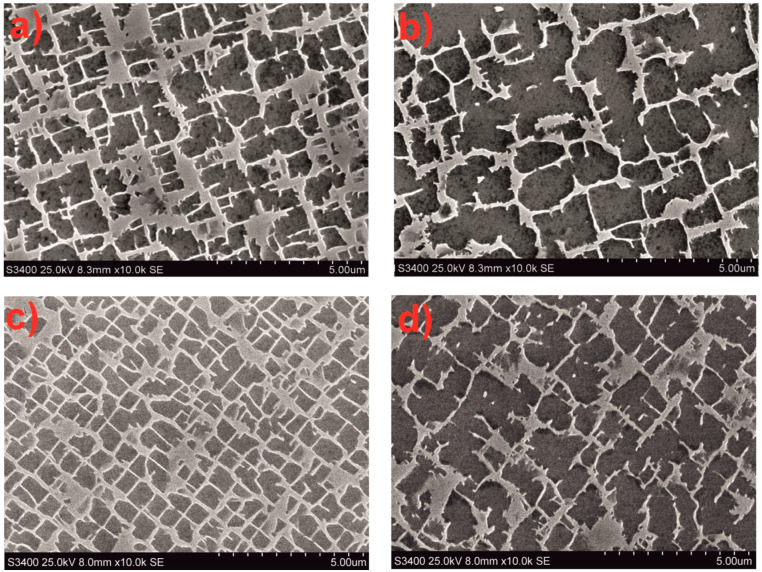
Microstructure of the CMSX-4 superalloy for the withdrawal rate: (**a**,**b**) 1, (**c**,**d**) 3 mm/min. Area: dendrite (**a**,**c**) and interdendritic region (**b**,**d**).

Investigations of the phase components of CMSX-4 single crystals in the as-cast state revealed that the γ’ phase crystallites have an irregular, cuboidal shape (Figure 8). The surface area of the γ’ phase crystallites showed differences in both size and content between dendritic and interdendritic regions. Larger γ’ crystallites were observed in the interdendritic regions compared to those in the dendritic regions (Table 3, Figure 8). As the withdrawal rate increased, the surface area of the γ’ phase crystallites decreased in both regions. The size differences of the γ’ phase crystallites in CMSX-4 single crystals ranged from 0.01 to 5.8 µm^2^.

**Figure 9:**
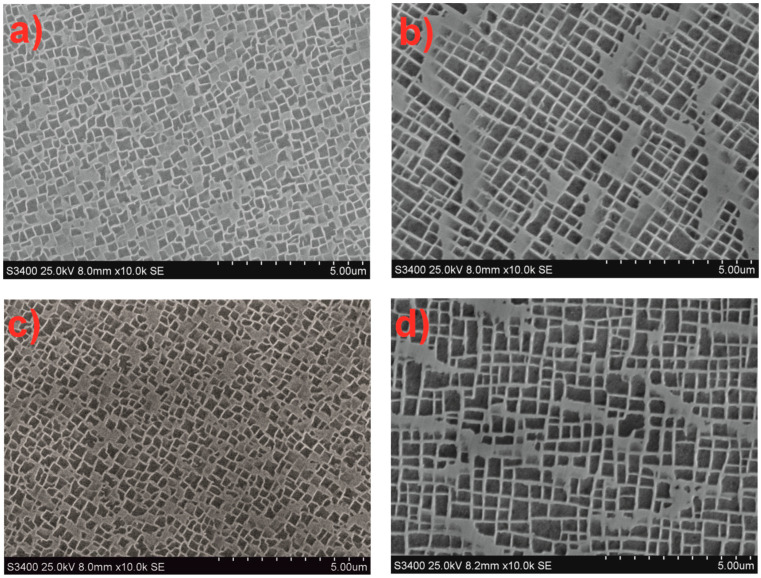
Microstructure of the CMSX-4 superalloy after heat treatment for the withdrawal rate: (**a**,**b**) 1, (**c**,**d**) 3 mm/min. Heat treatment, solution (**a**,**c**) and solution + 2 aging (**b**,**d**).

Scanning electron microscopy (SEM) investigations of CMSX-4 after solution heat treatment revealed the presence of secondary γ’ precipitates with a rosette-like morphology. Solution treatment also homogenized the morphology and size of the γ’ phase precipitates in both dendritic and interdendritic regions (Figure 9). The specific surface area of the γ’ phase crystallites was smaller after the solution treatment compared to the as-cast state.

It was further established that double aging after solution treatment resulted in a transformation of the secondary γ’ phase precipitates from a rosette shape to a cuboidal shape. These precipitates also increased in size after solution treatment and aging, compared to only the solution-treated state. The average surface area of the γ’ phase crystallites was found to be similar for w_r_ = 1 mm/min and w_r_ = 3 mm/min (Table 3). Therefore, it can be concluded that the heat treatment of CMSX-4 single crystals leads to the homogenization of their microstructure, regardless of the withdrawal rate.

### 3.2. Crystal Orientation

The crystallographic orientation, measured using the Ω-scan method, was characterized by analyzing three blades in the as-cast state, manufactured at a withdrawal rate (w_r_) of 1 mm/min. These blades were labeled A, B, and C–1 mm/min. Additionally, a blade produced at a w_r_ of 3 mm/min was also included in the study.

The values of the angles α_z_ (primary orientation), β_z_, and γ_z_ (secondary orientation) were determined with an accuracy of 0.01°, as described in the methodology section.

The primary orientation values for the blades manufactured at a w_r_ of 1 mm/min were as follows:

- Blade 1A: 7.9–9.7° (Figure 9a),
- Blade 1B: 6.8–8.2° (Figure 9b),
- Blade 1C: 4.8–6.2° (Figure 9c).

These results show that the blades exhibit a small deviation from the [001] crystallographic direction. Each blade, however, displayed a scatter in the αz angle, with a difference of 1.4° to 1.8° between the minimum and maximum values. An analysis of the primary orientation in the roots and airfoils of the blades manufactured at a w_r_ of 1 mm/min revealed no consistent trend for an increase in the α_z_ angle with the height of the casting (i.e., from the root to the airfoil as the crystallization front moves). Depending on the blade, the maximum values of αz occurred in the root (1B) or the airfoil (1A,C).

**Figure 10:**
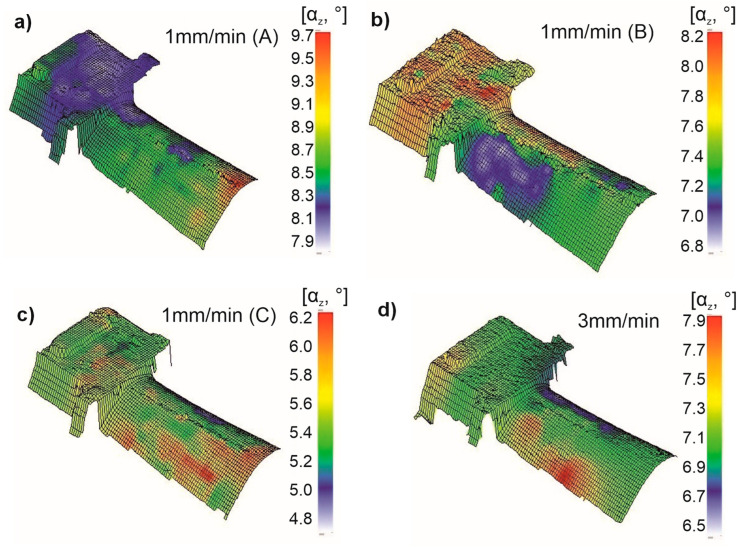
Distribution map of the deviation α_z_ angle values on the surface of the root and the airfoil of the NBSX blade casting made of CMSX-4 superalloy: (**a**–**c**)—1 mm/min, (**d**)—3 mm/min.

It was also observed that the airfoil exhibited greater scatter in the primary orientation values. For all three blades (1A–C), greater color differentiation on the maps indicates a larger scatter in the αz angle (Figure 10a–c).

The analysis of the primary orientation in the blade produced at a w_r_ of 3 mm/min yielded similar conclusions. The α_z_ angle on its surface ranged from 6.5° to 7.9° (Figure 10d), indicating similar deviations from the [001] direction and comparable differences between the minimum and maximum values as for the blades manufactured at 1 mm/min. A similar trend was observed, with greater scatter in the primary orientation values occurring in the airfoil region.

**Figure 11:**
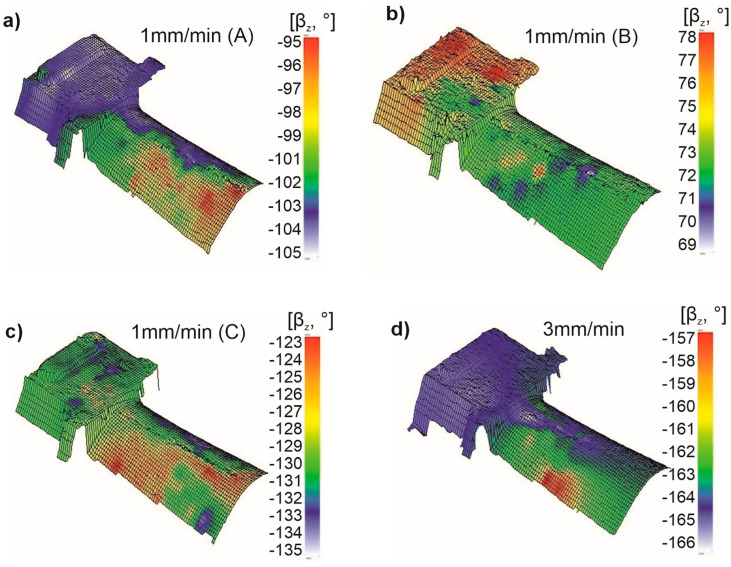
Distribution map of the deviation β_z_ angle values on the surface of the root and the airfoil of the NBSX blade casting made of CMSX-4 superalloy: (**a**–**c**)—1 mm/min, (**d**)—3 mm/min.

The β_z_ angle, which describes the rotation of the [001] direction around the z-axis (solidification direction), was also determined for the root and airfoil surfaces. The β_z_ angle ranges from −180° to +180°. In this analysis, the focus was on the scatter of β_z_ values across the surface rather than the specific values themselves. Blade 1A (w_r_ = 1 mm/min) exhibited a β_z_ scatter of 10° (from −95° to −105°) (Figure 11a). It was found that the β_z_ value remained constant across the root surface, while a larger scatter occurred in the airfoil. Blade 1B showed a similar overall β_z_ scatter of 9° (from 69° to 78°) (Figure 11b), but in this case, the greater scatter occurred in the root, opposite to the trend observed in blade 1A.

The largest β_z_ scatter was observed in blade 1C, with a variation of 14° (from −123° to −137°) (Figure 11c). In this blade, the scatter was slightly larger in the airfoil than in the root.

For the blade produced at a w_r_ of 3 mm/min, the β_z_ scatter was 9° (Figure 11d), similar to that of blade 1A. Greater misorientation occurred in the airfoil, while the root displayed an almost constant value, as shown by the uniform color in Figure 11d.

**Figure 12:**
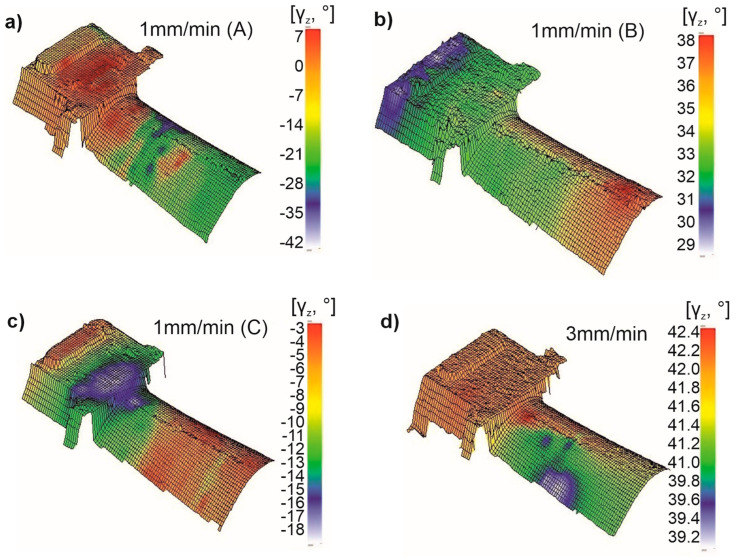
Distribution map of the deviation γ_z_ angle values on the surface of the root and the airfoil of the NBSX blade casting made of CMSX-4 superalloy: (**a**–**c**)–1 mm/min, (**d**)–3 mm/min.

To complete the crystallographic orientation characterization of the CMSX-4 single crystals, the γ_z_ angle was determined. The γ_z_ angle describes the deviation of the projection of the [010] direction from the second reference axis “y”. Similar to the analysis of the β_z_ angle, the focus was primarily on the scatter of the γz values. For blade 1A (w_r_ = 1 mm/min), the γ_z_ angle ranged from −42° to +7°, resulting in a total scatter of 49° across the surface (Figure 12a). Lower disorientation was observed in the root compared to the airfoil. The results for the secondary orientation of blade 1A indicated a similar trend to the β_z_ angle, with less scatter in the root and greater disorientation in the airfoil. Overall, the scatter of the γ_z_ angle across the entire blade surface was larger.

Blade 1B exhibited a much smaller scatter in γ_z_ values, ranging from 29° to 38°, with a total scatter of 9° (Figure 12b). The color distribution map suggests that the disorientation is similar in both the root and airfoil, despite changes in the γ_z_ angle along the height of the blade.

Similarly, for blade 1C, the γ_z_ angle ranged from −3° to −18°, resulting in a total scatter of 15° (Figure 12c). The distribution map indicated similar disorientation in both the root and airfoil regions.

The blade produced at a w_r_ of 3 mm/min exhibited the smallest γ_z_ scatter, with a total variation of 3.2° (values ranging from 39.2° to 42.4°) (Figure 12d). This blade showed the most consistent secondary orientation across both the root and airfoil surfaces. A more detailed analysis of the disorientation distribution indicated uniform color shading in the root, while a small disorientation of approximately 3° was observed in the airfoil.

### 3.3. Porosity

In this study, the porosity of the NBSX CMSX-4 superalloy was evaluated both in the as-cast state and after heat treatment. The porosity of single-crystal (SX) castings was measured on their cross-sections at a fixed distance from the base. The average porosity volume (V_a_) and maximum porosity volume (V_max_) were determined over a standardized surface area of 1.4 mm^2^.

**Figure 13:**
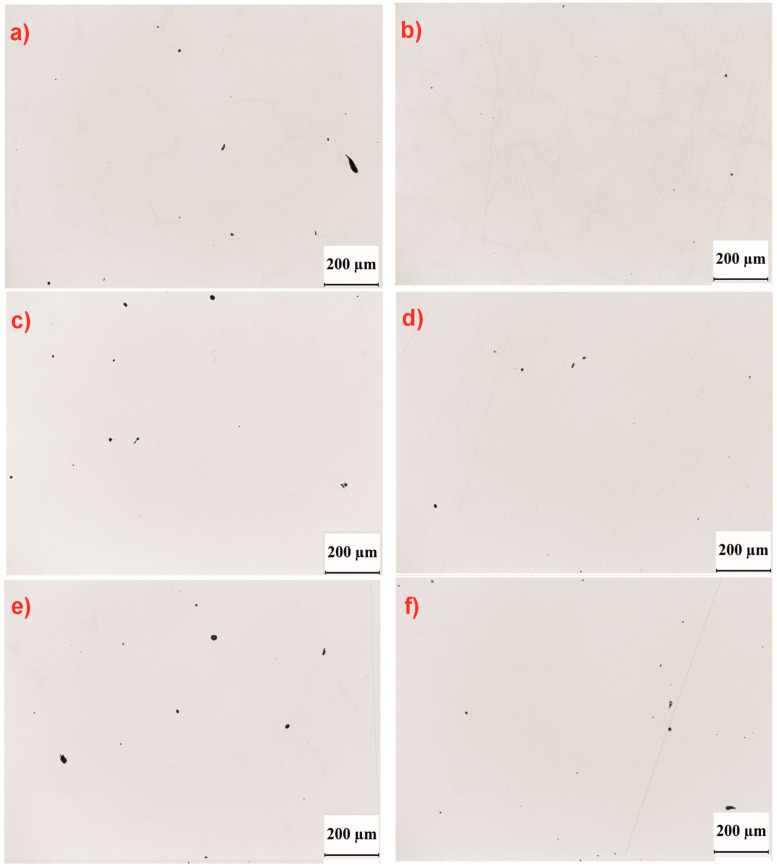
Surface of single-crystal cast nickel superalloy CMSX-4 with visible pores. Optical microscope, unetched sample: (**a**) as cast 1 mm/min, (**b**) as cast 3 mm/min, (**c**) solution 1 mm/min, (**d**) solution 3 mm/min, (**e**) solution + 2 aging 1 mm/min, (**f**) solution + 2 aging 3 mm/min.

In the as-cast condition, the highest levels of both average (V_a_) and maximum (V_max_) porosity were observed in the castings produced with a withdrawal rate (w_r_) of 1 mm/min (Table 4). The solution heat treatment resulted in an increase in porosity for castings produced at both withdrawal rates (1 and 3 mm/min). Additionally, the application of double aging further increased the porosity in the castings produced at 1 mm/min. For the withdrawal rate of 3 mm/min, the average porosity (V_a_) was comparable after solution treatment and aging (Table 4, Figure 13a–f). The largest pores, based on surface area, were found in the castings with a withdrawal rate of 1 mm/min.

### 3.4. Creep Resistance

The results of the creep tests revealed significant differences in the mechanical properties of the NBSX CMSX-4 superalloy, depending on the withdrawal rates (w_r_) during the solidification process and subsequent heat treatments.

**Figure 14:**
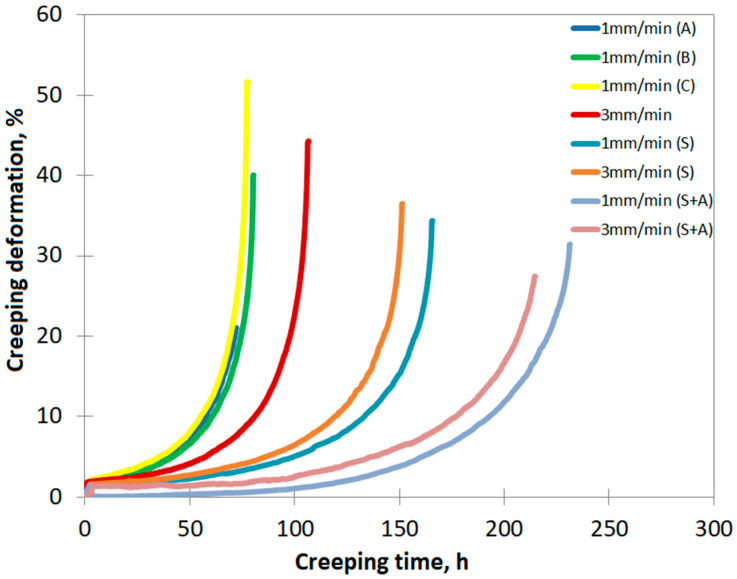
Creep curves of CMSX-4 superalloy depending on the withdrawal rate and heat treatment.

In the as-cast state, the castings produced at a withdrawal rate of 1 mm/min (1A–C) exhibited failure after similar durations: 1A—71 h, 1B—80 h, and 1C—77 h, respectively. In contrast, the casting produced at 3 mm/min failed after a slightly longer duration of 106 h (Figure 14).

The aging of the castings resulted in a significant improvement in creep resistance. For both withdrawal rates (1 and 3 mm/min), the results were comparable. The time to rupture for the solution-treated sample at 1 mm/min increased to 165 h, while for 3 mm/min, it reached 151 h. This suggests that solution treatment led to a more than twofold increase in creep resistance for the casting with a 1 mm/min withdrawal rate and an approximately 70% increase for the casting at 3 mm/min.

A similar enhancement in creep resistance was observed after full heat treatment (solution and double aging). The time to failure for the casting at 1 mm/min increased to 231 h, while for the 3 mm/min casting, it increased to 214 h. This represents a roughly threefold improvement in creep resistance for the casting at 1 mm/min due to heat treatment, while the casting at 3 mm/min experienced an approximately 2.5-fold improvement (Figure 14).

## 4. Discussion

The analysis of the results focused on two primary factors: the assessment of the blades in terms of their primary and secondary orientation values and the evaluation of selected microstructure parameters. Additionally, the influence of selected crystal structure and microstructure characteristics on strength properties, particularly creep resistance, was examined.

The blade castings produced at withdrawal rates (w_r_) of 1 and 3 mm/min using the Bridgman–Stockbarger method exhibited a dendritic structure, with secondary dendrite arm spacing (SDAS) values typical for such castings. An increase in the withdrawal rate led to a decrease in SDAS (Table 1) [26,27]. The volume fractions of the γ and γ’ phases were within the expected range (Table 2) [28]. At the same time, a decrease in the withdrawal rate resulted in an increase in γ’ precipitates, particularly in the interdendritic regions (Table 3) [29]. Heat treatment, including solution and double aging, led to an increase in the γ’ phase volume by approximately 2–4%, with a more pronounced increase observed at the higher withdrawal rate of 3 mm/min. Additionally, the secondary γ’ phase precipitates became more cubic in shape and reduced in size (Figure 8 and Figure 9) [30]. The primary orientation of the manufactured blades did not exceed 10°, a critical threshold beyond which blades are typically rejected from production [31,32]. However, the withdrawal rate did not significantly affect the primary orientation, as results were similar for both 1 and 3 mm/min rates. The distribution of the primary orientation was found to be random, varying between the root and airfoil of the blade (Figure 10).

The issue of primary orientation dispersion is complex, influenced by the blade’s geometry, which is determined by design and technological constraints. This problem is difficult to address, particularly considering the requirements for single-crystal material technologies. However, it can be suggested that achieving a minimal deviation angle (α_z_) and reduced orientation scatter can be facilitated by optimizing the design of the starter and selector in the ceramic mold. According to the Bridgman–Stockbarger method, these components should promote the growth of a single nucleus, resulting in minimal disorientation within the blade.

Regarding secondary orientation, no data are available in the literature for a direct comparison of its distribution. It has been assumed that the secondary orientation should remain as consistent as possible throughout the blade casting [33]. Hence, the results were not compared to external data, but it was established that the scatter of secondary orientation ranged from about 3° to nearly 50°, a very wide variation that warrants reduction. Based on the obtained results, it was observed that a more uniform primary orientation correlated with reduced scatter in secondary orientation and the β_z_ angle (Figure 10d, Figure 11d and Figure 12d). Therefore, optimizing the primary orientation during production is essential to improving secondary orientation consistency, ultimately enhancing the blade’s performance. This goal can be achieved by refining the technological process of blade production to minimize the primary orientation deviation, which, in turn, will stabilize the secondary orientation [13,29].

**Figure 15:**
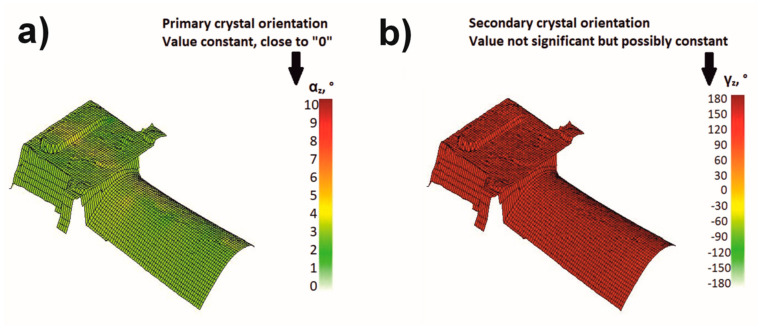
Blade model with predicted crystallographic orientations that can lead to high creep resistance: (**a**) primary crystal orientation, (**b**) secondary crystal orientation.

In the summary, a blade model is presented where one should strive to obtain primary and secondary crystallographic orientations. The primary crystallographic orientation is close to the [001] direction, and the deviation is about 2–3° on the blade surface. The secondary orientation is also close to constant, and the angle is about 180° (Figure 15).

When comparing the crystallographic orientation results with creep strength, drawing clear conclusions is challenging due to the limited number of creep tests (only four) performed in the as-cast state and the assumption that the blade orientation matches the rod’s orientation (the creep sample). However, it was found that the casting produced at 3 mm/min demonstrated better creep resistance than that at 1 mm/min (Figure 14). Factors contributing to this difference may include a smaller dispersion in secondary crystallographic orientation, smaller SDAS, finer γ’ phase precipitates, and lower porosity (Table 4). The primary orientation was similar for both withdrawal rates. Therefore, with comparable primary orientation, the differences in creep test failure times could be attributed to other parameters, often not evaluated during routine blade production control [34].

Solution heat treatment and aging resulted in creep curves with similar times to failure, with slightly longer times observed for the 1 mm/min withdrawal rate. Although the 3 mm/min casting showed superior crystal structure and microstructure parameters in the as-cast state, the creep test results were comparable. This suggests that further research should focus on the effect of secondary orientation on the creep strength of single-crystal castings after heat treatment.

The optimization of the ceramic mold’s starter and selector design can promote single-grain growth and minimize primary orientation deviation, ensuring better mechanical performance.

Stabilizing the secondary orientation through controlled withdrawal rates or seed casting can further reduce the risk of stress concentrations at grain boundaries, enhancing fatigue and creep resistance.

While the Ω-scan method provides comprehensive surface-wide data, it does not account for subsurface defects, which may require complementary techniques such as transmission Laue diffraction for complete analysis.

## 5. Conclusions

The analysis of the obtained results leads to the following conclusions:

1. Blade castings produced at withdrawal rates of 1 and 3 mm/min exhibited similar primary orientation; however, the secondary orientation showed significant variation between the root and airfoil surfaces.
2. Creep tests of as-cast castings revealed that the casting with the least dispersion in the secondary orientation demonstrated the longest time to failure during the creep test.
3. Secondary crystal orientation showed the greatest influence on the creep resistance of castings in the as-cast state, whereas after heat treatment they showed the lowest, with an applied withdrawal rate of 1 mm/min.
4. Heat treatment of the NBSX CMSX-4 superalloy resulted in a 2.3- to 3-fold increase in creep resistance compared to the as-cast condition.
5. To improve the performance of blade castings, it is essential to aim for minimal deviation and a consistent value of primary orientation across both the root and airfoil, as this also influences the uniformity of secondary orientation.
6. The findings suggest that introducing seed grains during the Bridgman–Stockbarger process could further enhance control over primary and secondary orientations, offering potential scalability for advanced single-crystal superalloy production in the aerospace industry.

## Figures and Tables

**Table 1:** Secondary dendrite arm spacing values.

Withdrawal Rate w_r_, mm/min	Secondary Dendrite Arm Spacing (Standard Deviation) (Δλ_2_), μm
1	97 (7)
3	72 (4)

**Table 2:** Relative volume of γ’ phase crystallites in the microstructure of NBSX CMSX-4 superalloy.

Withdrawal Rate mm/min	As Cast			Solution	Solution + Double Aging
	The Average Relative Volume of γ’ Phase	In Dendrite	Interdendritic Region		
1	64.8 (8.4)	55.9 (1.0)	69.2 (6.2)	67.3 (4.8)	67.2 (5.1)
3	64.0 (6.8)	57.8 (1.0)	68.1 (5.2)	65.0 (5.2)	68.0 (3.0)

**Table 3:** Surface area of a plane cross-section of γ’ phase crystallites in the NBSX CMSX-4 superalloy.

Withdrawal Rate mm/min	As Cast						Solution			Solution + Double Aging		
	In Dendrite			Interdendritic Region								
	Min	Max	Ave	Min	Max	Ave	Min	Max	Ave	Min	Max	Ave
1	0.01	1.94	1.36	0.01	5.8	2.26	0.02	0.95	0.11	0.04	1.22	0.14
3	0.01	1.56	1.22	0.01	4.3	1.8	0.03	0.76	0.08	0.04	1.02	0.12

**Table 4:** Relative volume of pores in the microstructure of NBSX CMSX-4 superalloy in the as-cast state and after heat treatment.

Withdrawal Rate w_r_, mm/min	As Cast			Solution			Solution + Double Aging		
	V_a_, %	σ, %	V_max_, %	V_a_, %	σ, %	V_max_, %	V_a_, %	σ, %	V_max_, %
1	0.079	0.021	0.283	0.085	0.024	0.336	0.116	0.071	0.894
3	0.024	0.012	0.147	0.049	0.016	0.207	0.047	0.007	0.093

## Data Availability

The original contributions presented in the study are included in the article, further inquiries can be directed to the corresponding author.
